# Effects of Plant and Soil Characteristics on Phyllosphere and Rhizosphere Fungal Communities During Plant Development in a Copper Tailings Dam

**DOI:** 10.3389/fmicb.2020.556002

**Published:** 2020-09-29

**Authors:** Tong Jia, Yushan Yao, Tingyan Guo, Ruihong Wang, Baofeng Chai

**Affiliations:** Shanxi Key Laboratory of Ecological Restoration on Loess Plateau, Institute of Loess Plateau, Shanxi University, Taiyuan, China

**Keywords:** plant and soil characteristics, phyllosphere and rhizosphere, fungal community, plant development, copper tailings dam

## Abstract

Interactions between plants and microbes can affect ecosystem functions, and many studies have demonstrated that plant properties influence mutualistic microorganisms. Here, high-throughput sequencing was used to investigate rhizosphere and phyllosphere fungal communities during different plant development stages. Results demonstrated that phyllosphere and rhizosphere fungal community structures were distinct during all developmental stages while they were mediated separately by plant carbon and soil sulfur. Comparatively, the effect of root properties on phyllosphere fungal diversity was greater than soil properties. Moreover, rhizosphere fungal networks of *Bothriochloa ischaemum* were more complex than phyllosphere fungal networks. This study demonstrated that the effect of plant and soil traits on phyllosphere and rhizosphere fungal communities could potentially be significant, depending on the applicable environmental condition and plant development stage. Although links between phyllosphere and rhizosphere communities have been established, further studies on functional fungal groups during phytoremediation processes are necessary. This study comprehensively analyzed dynamic relationships between phyllosphere and rhizosphere fungal communities during different plant development stages in a polluted environment. These fungal communities were determined to be expedient to the development and utilization of beneficial microbial communities during different development stages, which could more effectively help to stabilize and reclaim contaminated copper tailings soil.

## Highlights

- Phyllosphere and rhizosphere fungal community structures were distinct during all stages of development.
- Plant carbon and soil sulfur separately mediated phyllosphere and rhizosphere fungal communities.
- Comparatively, the effect of root properties on phyllosphere fungal diversity was greater than soil properties.

## Introduction

Phyllosphere and rhizosphere microbial communities are significant components of plant microbiota (Zhu et al., 2019). Phyllosphere microbes colonize aerial components of plants or inhabit plant tissues and organs, including caulosphere, anthosphere, carposphere, and phylloplane (Meyer and Leveau, 2012; Vorholt, 2012; Zarraonaindia et al., 2015). Globally, the leaf area of plants is estimated to be 10^9^ km^2^, which is almost twice that of global land area (Vorholt, 2012). Moreover, it is estimated that phyllosphere bacterial abundance may exceed 10^62^ cells globally (Lindow and Brandl, 2003). At present, even though data on phyllosphere fungal abundance are lacking, it is estimated to be much lower than that of bacteria (Zhu et al., 2019). Phyllosphere microbes mainly derive from indigenous plant microorganisms as well as exogenous microorganisms transported by means of horizontal propagation through soil and air or by other plants (Müller et al., 2016). The rhizosphere is the shallow soil region of belowground plant components that is directly affected by roots and root secretions (i.e., root exudates). Rhizosphere microbes mainly derive from the soil environment (Mendes et al., 2013). The abundance of the rhizosphere microbial community far exceeds that of the host plant, reaching 10^11^ cells/root (Mendes et al., 2011).

Phyllosphere microbes participate in a variety of functions, which play key roles in nitrogen fixation (Fürnkranz et al., 2008), promoting plant growth (Bulgarelli et al., 2013; Rastogi et al., 2013), inhibiting pathogenic microorganisms (Rastogi et al., 2013), and degrading environmental pollutants (Yutthammo et al., 2010); however, these microbes also cause negative effects to occur, such as plant diseases. Studies have shown that plant species, host plant genotypes (Costa et al., 2012; Kim et al., 2012), seasonal changes, geographical locations (Redford et al., 2010; Finkel et al., 2011; Rastogi et al., 2012), and the environment itself (Gu et al., 2010; Sun et al., 2018) can affect phyllosphere microbial community characteristics. Moreover, leaf properties also significantly affect microbial community structure (Whipps et al., 2008). For example, different plant leaf characteristics, such as those associated with the stomata, water glands, leaf thickness, and nutrient and water content, affect phyllosphere microbial colonization (Yadav et al., 2005; Kembel and Mueller, 2014; Kembel et al., 2014). Previous studies have also shown that the phyllosphere is very complex (Hooker et al., 2007). The effects of environmental and biological factors on phyllosphere microbial community composition remain unclear when all these factors are considered together. Although rhizosphere microbes promote plant growth, being closely correlated to both plant growth and health, some of these microbes also inhibit plant growth. Rhizosphere microbial community composition differs among plant species, plant development stage, and other factors (Zhou et al., 2016). It has been reported that changes to rhizosphere microbial communities are affected by plant development (Houlden et al., 2008; Micallef et al., 2009; Chaparro et al., 2014). Xu et al. (2009) found that more complex microbial communities were found during the early reproductive growth stages of *Glycine max* than during its latter stages. Notably, a study conducted on the core microbiome of *Arabidopsis thaliana* can be used as a tool to determine the plant’s influence on its mutualistic rhizosphere microbiome during developmental stages (Lundberg et al., 2012). Therefore, microbial community structure must be assessed through plant developmental stages, which focus on members that comprise the microbial community.

The copper mine selected for this study, the Northern Copper Mine, is the largest underground copper mine in China. It has an annual output of greater than 7 million tons of ore. A large amount of heavy metals are disposed directly into soil (Jia et al., 2019). Such activities not only lead to the considerable degradation of soil ecosystems, but also affect plant growth and development. In our previous study, *B. ischaemum* (L.) Keng is the dominant plant species in this tailings dam, and their infected fungal species had phytoremediation potential in mining area (Jia et al., 2017, 2018b,c). Moreover, both rhizosphere and phyllosphere microbial activities are correlated to plant microbes, forming a unique microecosystem within the broader environment. Therefore, clarifying compositional and dynamical phyllosphere and rhizosphere microbiome characteristics is key to understanding how these communities affect the health and development of plants. Many studies have focused on fungal community respective of plants in natural ecosystem (Sánchez Márquez et al., 2011; Jia et al., 2018c), while fungal community dynamics during plant development in the damaged ecosystem remain elusive. It is critical that we clarify the influence of plant and soil traits on fungal community structure during plant development, which will also contribute to the generation of more healthy and resistant seedlings. Accordingly, this study investigated the fungal community structure and function associated with the phyllosphere and rhizosphere of *B. ischaemum* during three different plant development stages: seedling, tiller, and mature. This study aimed (i) to examine dynamic relationships associated with phyllosphere and rhizosphere fungal communities during plant development and (ii) to test whether driving factors associated with these phyllosphere and rhizosphere fungal communities differ.

## Materials and Methods

### Site Description

Construction on the Shibahe copper tailings dam (latitude 35°15' ~ 35°17'N and longitude 118°38' ~ 111°39'E) commenced in 1969, which is a part of the broader Northern Copper Mine, situated in the southern region of China’s Shanxi Province. In total, this particular dam comprised of 16 sub-dams. The study area is marked by four distinct seasons subjected to a continental “monsoon” climate, wherein annual mean temperature = 14°C, annual precipitation = ~780 mm, and frost free days = > 200 (Jia et al., 2017).

### Plant and Soil Sampling

The No. 536 sub-dam of the Shibahe copper tailings dam was selected for study in 2017. This sub-dam, in its 20 years of restoration, was sampled during three predetermined plant growth stages during the early of June and the middle of July and September (i.e., seedling, tiller, and mature). The dominant plant species in this sub-dam was *B. ischaemum*. We randomly collected leaves from *B. ischaemum* samples as well as rhizosphere soil samples in three 1 × 1 m sample plots, where each sample plot was spaced greater than 50 m apart. In each plot, 60 leaf samples were selected. These leaf samples were sealed in sterile plastic bags using tweezers sterilized in ethanol. For the plant samples, one subsample was used to determine physiochemical properties, and the other was transported to the laboratory, where it was stored (−20°C) in advance of high-throughput sequencing. For the rhizosphere soil samples, we first removed visible roots as well as any residue before each sample’s soil fraction was homogenized. The sterile gloves should be worn throughout the sampling process to avoid contamination of the samples. We then divided the fresh soil samples into two subsamples after being sifted (using a 2 mm sieve). We stored the first subsample (4°C) to determine physiological and chemical properties, while we stored the second subsample (−20°C) to extract DNA.

### Plant and Soil Chemical Properties

An elemental analyzer (vario EL/MACRO cube, Elementar, Hanau, Germany) was used to measure total carbon (TC), total nitrogen (TN), and total sulfur (TS) content in plant and soil samples (Supplementary Tables S1 and S2). Soil water (1:2.5 mass/volume) suspensions were shaken for 30 min prior to measuring soil pH. Gravimetric analysis was used to measure soil moisture. An automatic discrete analyzer (CleverChem 380, DeChem-Tech, GmbH, Hamburg, Germany) was then used to measure soil constituents, namely, ammonium nitrogen (NH_4_^+^-N), nitrate nitrogen (NO_3_^−^N), and nitrite nitrogen (NO_2_^−^-N; Supplementary Table S2).

### Techniques Used for DNA Extraction, PCR Amplification, and MiSeq Sequencing

Leaf samples were washed three times in a sterile phosphate buffer solution (PBS: NaCl, KCl, Na_2_HPO_4_, and KH_2_PO_4_) prior to filtering through means of a sterile membrane filter (0.2 μm pore size; Millipore, Jinteng, Tianjin, China). The filtered samples used to extract microbial DNA were sealed in sterile centrifuge tubes. Microbial DNA taken from plants and soil was extracted using the E.Z.N.A.® Soil DNA Kit (Omega Bio-tek, Norcross, GA, United States) under the manufacturer’s protocol. The NanoDrop ND-1000 UV-Vis spectrophotometer (NanoDrop Technologies, Wilmington, DE, United States) was used to quantify extracted DNA. Primers ITS1F (5'-CTTGGTCATTTAGAGGAAGTAA-3') and ITS2 (5'-GCTGCGTTCTTCATCGATGC-3') were used as the fungal ITS gene copy numbers of all samples. We carried out sequencing at Shanghai Majorbio Bio-pharm Technology (Shanghai, China) using the MiSeq platform (Illumina, Inc., CA, United States). Finally, we submitted raw sequencing data to the National Center for Biotechnology Information (NCBI) Sequence Read Archive (SRA; https://www.ncbi.nlm.nih.gov/sra) under the project accession number PRJNA605500.

### Processing of Sequencing Data

Raw FASTQ files were demultiplexed and quality-filtered using QIIME (version 1.17) under the following criteria: 300-bp reads were truncated at any site receiving an average quality score of <20 over a 50-bp sliding window, and truncated reads shorter than 50 bp were discarded; exact barcode matching, two-nucleotide mismatch in primer matching, and reads containing ambiguous characters were removed; and only sequences that overlapped for more than 10 bp were merged according to their overlap sequence. Reads that could not be merged were discarded. Operational taxonomic units (OTUs) were clustered with a 97% similarity cutoff using UPARSE (version 7.1; http://drive5.com/uparse/), and chimeric sequences were identified and removed using UCHIME. The taxonomy of ITS gene sequences were analyzed using the Ribosomal Database Project (RDP) Classifier^1^ against the unite 7.0 ITS fungi database with a confidence threshold of 70%.

### Statistical Analysis

SPSS Statistics version 20 was used to calculate the data derived from the above analyses. Heatmapping of the top 10 genera in each sample was conducted using the R packages. Canoco 5.0 (Microcomputer Power, United States) was used for non-metric multidimensional scaling (NMDS) analysis and redundancy analysis (RDA) to investigate relationships among fungal and environmental factors. One-way ANOVA was used to detect any disparities in environmental parameters, alpha diversity (α-diversity) indexes as well as the relative species abundance of the dominant fungal species among the three selected plant growth stages (i.e., seedling, tiller, and mature). Additionally, the spearman’s rank correlation test was used to ascertain any correlations among fungal communities and environmental variables. Linear discriminant analysis Effect Size (LEfSe) analysis was used to detect biomarkers that were statistically different among groups (Segata et al., 2011). The interactive Gephi platform was used to explore network properties and visualize networks (Bastian et al., 2009). Spearman’s correlation was used to generate association networks. The nodes in the networks represent fungal families, and the edges connecting these nodes represent correlations between fungal families in relative abundances. Network properties were calculated with the igraph package in R. The topological properties include the number of nodes, the number of edges, average degree, network diameter, clustering coefficient, average path length, and betweenness centrality. AMOS 13.0 was used to analyze structural equation models (SEM). The Fungi Functional Guild (FunGuild; Nguyen et al., 2016), an open annotation tool that has recently been developed, was used to ascertain distinct functional groups within the fungal communities.

## Results

### Overall Taxonomic Distribution and Fungal Diversity

**Figure 1:**
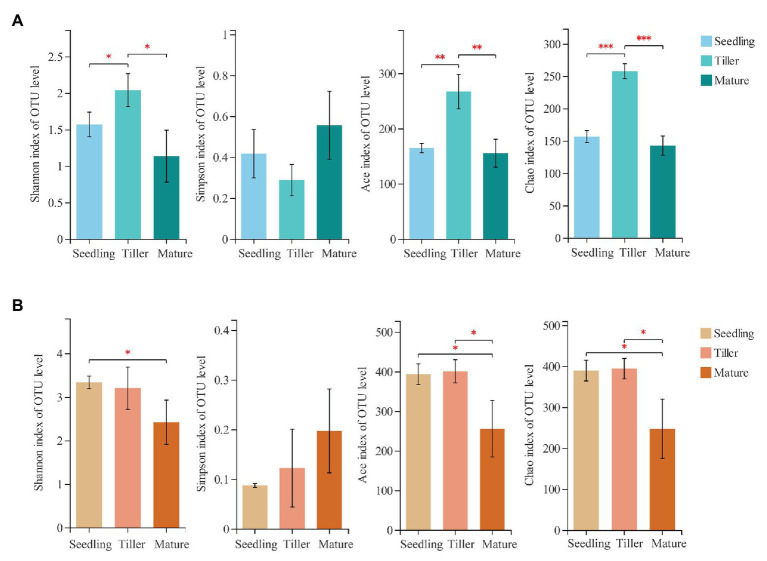
Estimated values of phyllosphere **(A)** and rhizosphere **(B)** fungal community relative abundances and diversity index. Asterisks indicate significant differences among plant development stages (Student’s *t*-test for estimator, ^*^p < 0.05; ^**^p < 0.01; ^***^p < 0.001).

Across all samples, we obtained a total of 6,87,098 high-quality sequence reads whose average length was 265 bp. Moreover, 375 fungal OTUs from phyllosphere and 693 fungal OTUs from rhizosphere soil samples were determined based on 97% sequence similarity, which indicated that the sequencing data reflected most fungal diversity in the field. Taxonomically classified OTUs were all associated with six phyla, 24 classes, 62 orders, 127 families, and 225 genera in the rhizosphere soil samples. For the phyllosphere samples, corresponding taxonomically classified OTUs were associated with five phyla, 20 classes, 46 orders, 90 families, and 163 genera. Richness (Ace and Chao1) and diversity (Shannon and Simpson) index values of the phyllosphere and rhizosphere fungal communities revealed that dynamic change occurred during the three plant development stages (Figure 1). The Shannon index and phyllosphere fungal community richness were both significantly higher during the tiller stage compared to the other two stages (Figure 1A). For the rhizosphere, fungal community richness was greater during the seedling and tiller stages compared to the mature stage, and the Shannon index was significantly higher during the seedling stage than the mature stage (Figure 1B).

### Fungal Community Comparison Among Plant Development Stages

**Figure 2:**
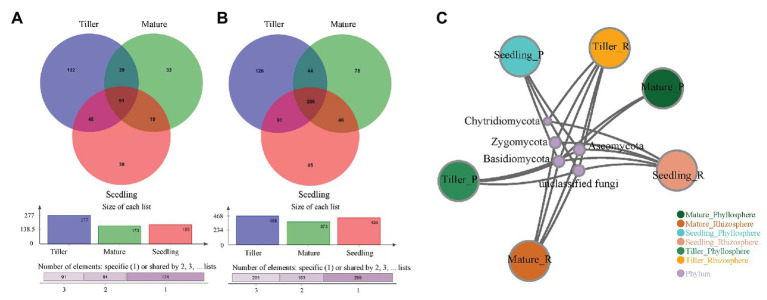
Venn diagram for phyllosphere **(A)** and rhizosphere **(B)** fungal communities and the network analysis on phylum level **(C)** among plant development processes. Numbers indicated shared unique operational taxonomic units (OTUs) at 0.03 dissimilarity distances after removing singletons involved.

The Venn diagram revealed that 91 OTUs were common to all phyllosphere fungal communities, while 205 were common to all rhizosphere fungal communities (Figure 2). Rhizosphere fungal communities yielded a greater number of unique OTUs compared to phyllosphere fungal communities during each plant development stage (Figures 2A,B). Moreover, the shared fungal phyla were Ascomycota, Basidiomycota, Zygomycota, and Chytridiomycota between the phyllosphere and rhizosphere (Figure 2C). Ascomycota and Basidiomycota were the dominant fungal phyllosphere phyla found in samples. Ascomycota, Basidiomycota, and Zygomycota were the dominant fungal rhizosphere phyla found in samples, and their relative abundance varied among plant growth stages. During the seedling stage, Basidiomycota exhibited significantly higher relative abundance compared to the tiller and mature stages, while Ascomycota exhibited the opposite effect. Ascomycota increased from 78.0% (seedling) to 94.6% (mature), and Basidiomycota decreased from 7.4% (seedling) to 0.7% (mature) in the rhizosphere (Supplementary Figure S1). Pleosporaceae was the dominant fungal family found in phyllosphere samples, and its members were present in greater than 48.5% of fungal sequences during plant development stages (Supplementary Figure S1). The maximum percent of Sporormiaceae was 30.5% at the seedling stage (Supplementary Figure S1). In contrast, Herpotrichiellaceae were the dominant fungal family found in rhizosphere samples, while Lophiostomataceae had the highest value (47.5%) during the mature stage (Supplementary Figure S1). Bray-Curtis dissimilarity analysis based on NMDS and ANOSIM was conducted to determine dissimilarity within fungal communities among each plant development stage. ANOSIM revealed significant differences in the fungal community structure of both the phyllosphere (stress = 0.045; *R* = 0.4733; *p* = 0.001) and rhizosphere (stress = 0.033; *R* = 0.9752; *p* = 0.001) among the different plant growth stages (Supplementary Figure S2).

**Figure 3:**
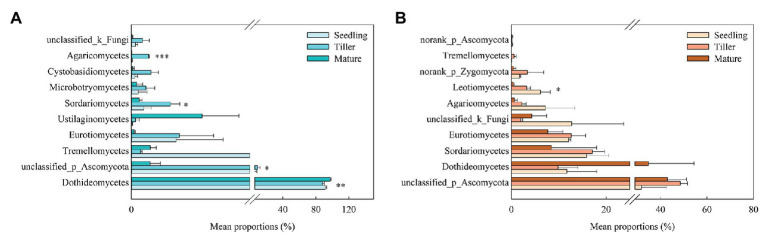
Relative abundances of top 10 fungal classes that showed significant differences among phyllosphere **(A)** and rhizosphere **(B)** samples from the seedling, tiller, and mature stages. A one-way ANOVA was used to evaluate the significance of differences between the indicated groups. ^*^p < 0.05; ^**^p < 0.01; ^***^p < 0.001.

The top 10 dominant classes are shown in Figure 3. For the phyllosphere fungal community, we found significant differences in Dothideomycetes, unclassified class of Ascomycota, Sordariomycetes, and Agaricomycetes at a class level among the plant development stages (Figure 3A). For the rhizosphere fungal community, only Leotiomycetes exhibited significant differences at a class level among the plant development stages (Figure 3B). Cladograms were used to depict groups, and LEfSe was used to confirm linear discriminant analysis (LDA) scores of 2 or greater (Supplementary Figure S3). During the seedling stage, two phyllosphere fungal groups were found to be significantly enriched, namely, Phaeosphaeriaceae (from a family to a genus level) and Mycosphaerellaceae (from a family to a genus level; Supplementary Figure S3). Fewer phyllosphere fungi were significantly enriched during the tiller stage, except for Ustilaginales (from an order to genus level). During the mature stage, eight phyllosphere fungal groups were highly enriched, while no rhizosphere fungal groups were found to be so (Supplementary Figure S3).

### Relationships Among Fungal Community Structure and Plant and Soil Characteristics

**Figure 4:**
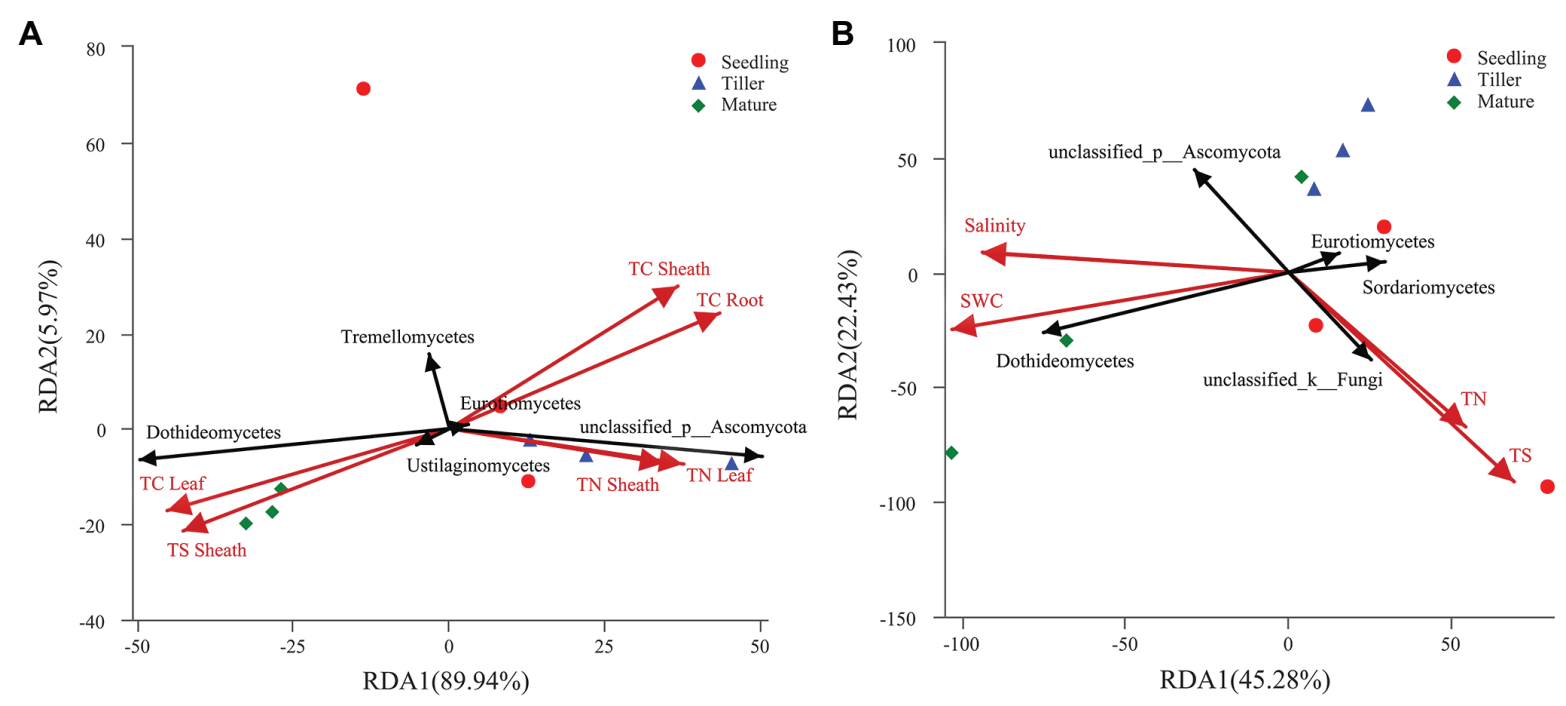
Redundancy analysis (RDA) of the top five fungal classes and leaf or soil characteristics. Phyllosphere and rhizosphere fungal communities are shown in **(A,B)**, respectively. The values of axes 1 and 2 are the percentages explained by the corresponding axis.

The leaf and soil properties showed that leaf TN was highest, but soil TN was lowest in tiller stage (Supplementary Tables S1 and S2). Both leaf TC and sheath TS were higher in mature stage than other stages (Supplementary Table S1). Soil nutrients (TC, TN, and TS) were highest in seedling stage, while soil NH_4_^+^-N and NO_2_^−^-N were highest in mature stage (Supplementary Table S2). Our experiment evaluated the effect of ecological factors on the top five fungal classes in phyllosphere and rhizosphere communities using RDA. We found correlations among plant and soil fungal community structure and characteristics (Figure 4). Results showed that 95.91% of variation in phyllosphere fungi could be explained by leaf properties (Figure 4A). Moreover, axis 1 of the RDA plot was able to explain roughly 89.94% of variation, while axis 2 was able to explain a further 5.97%. Results showed that leaf TC (*p* = 0.007), sheath TC (*p* = 0.005), root TC (*p* = 0.002), and sheath TS (*p* = 0.003) significantly affected phyllosphere fungal community structure (Figure 4A). Soil properties were able to explain 67.71% of variability in rhizosphere fungal community structure (Figure 4B), wherein axis 1 of the RDA plot explained 45.28% of variability, and axis 2 explained a further 22.43%. Four soil characteristics were chosen for RDA after redundant variables were removed. As shown in Figure 4B, soil TS (*p* = 0.009) significantly affected rhizosphere fungal community structure.

**Figure 5:**
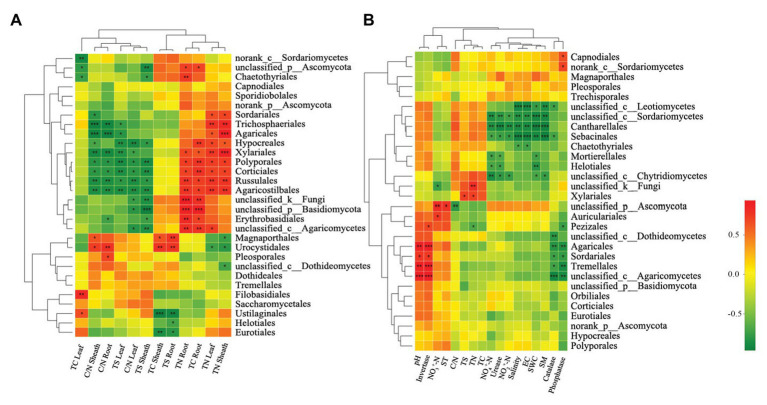
Correlation heatmap of the top 30 orders and soil properties and vegetation. Phyllosphere and rhizosphere fungal communities are shown in **(A,B)**, respectively. X and Y axis are environmental factors and orders. *R* in different colors to show, the right side of the legend is the color range of different *R* values. ^*^p < 0.05; ^**^p < 0.01; ^***^p < 0.001.

Microbiota structure is typically shaped by both abiotic and biotic environmental variables (as seen in Figure 5). The correlation heatmap showed that relationships among fungal orders and plant properties or environmental factors differed between the phyllosphere and rhizosphere (Figure 5). For the phyllosphere, Hypocreales, Xylariales, Polyporales, Corticiales, Russulales, Agaricostilbales, norank_c_Dothideomycetes, Cantharellales, Trichosphaeriales, Agaricales, and Sordariales exhibited significant positive correlations to plant nitrogen (including leaf TN and sheath TN). Moreover, Hypocreales, Xylariales, Polyporales, Corticiales, Russulales, Agaricostilbales, and norank_c_Dothideomycetes exhibited extremely significant negative correlations to leaf and sheath TS (Figure 5A). For the rhizosphere fungal community, Agaricales, Sordariales, Tremellales, and unclassified order of Agaricomycetes abundance were extremely positively correlated to pH, while unclassified order of Sordariomycetes, Cantharellales, Sebacinales, Mortierellales, Helotiales, and unclassified order of Chytridiomycetes abundance was significantly negatively correlated to soil NH_4_^+^-N and SWC. Furthermore, Xylariales was significantly and positively correlated to soil TN and TS, while Pezizales was significantly and negatively correlated to TN (Figure 5B).

**Figure 6:**
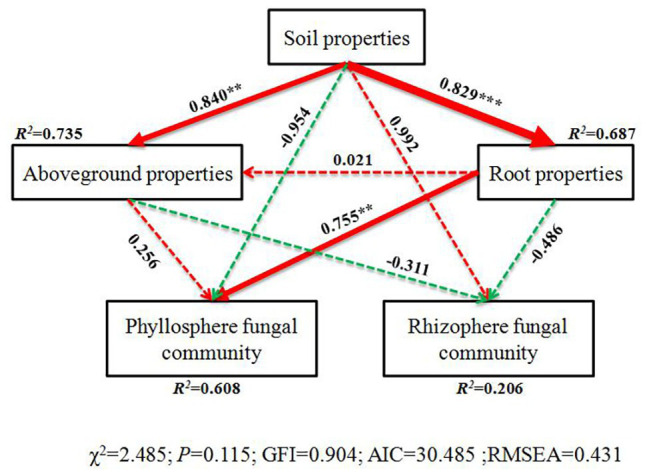
Structural equation model (SEM) illustrating the effects of soil properties on physicochemical characteristics (aboveground and root) and fungal communities of phyllosphere and rhizosphere. Continuous and dashed arrows represent the significant and non-significant relationships, respectively. Adjacent numbers that are labeled in the same direction as the arrow represents path coefficients, and the width of the arrow is in proportion to the degree of path coefficients. Green and red arrows indicate positive and negative relationships, respectively. *R*^2^ values indicate the proportion of variance explained by each variable. Significance levels are denoted with ^**^p < 0.01 and ^***^p < 0.001. Standardized total effects (direct plus indirect effects) calculated by the SEM are displayed below the SEM. The low chi-square (*χ*^2^), nonsignificant probability level (*p* > 0.05), high goodness-of-fit index (GFI > 0.90), low Akaike information criteria (AIC), and low root-mean-square errors of approximation (RMSEA < 0.05) listed below the SEMs indicate that our data match the hypothetical models.

**Figure 7:**
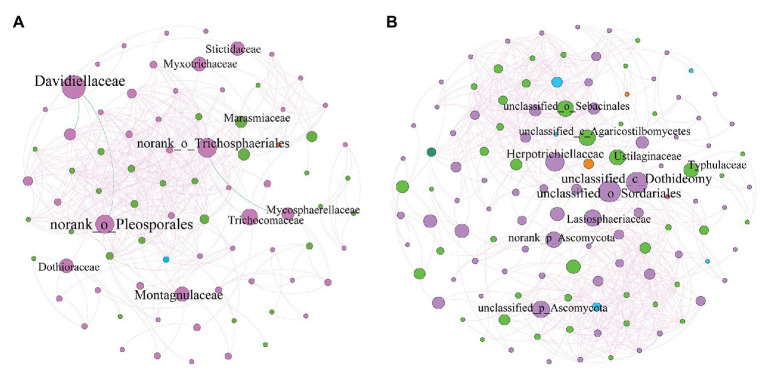
Co-occurrence network of microbial taxa on phyllosphere **(A)** and rhizosphere **(B)**. Nodes represent fungi families, whereas pink and green edges respectively represent positive and negative connections between pairs of species.

We constructed the SEM to further quantify the contribution of each potential influential factor (including soil, aboveground, and root properties) to the fungal community beta diversity (β-diversity) of the phyllosphere and rhizosphere (Figure 6). Root properties were the dominate factors that influenced phyllosphere fungal diversity. Soil properties indirectly affected phyllosphere fungal diversity, while we observed no impact associated with plant development processes on the rhizosphere fungal community (Figure 6). Furthermore, our study also provides co-occurrence networks of fungal taxa on the phyllosphere and rhizosphere (Figure 7 and Supplementary Table S3). The phyllosphere fungal network in this study consisted of 82 nodes and 416 edges, and the network diameter and clustering coefficient were 9 and 0.685, respectively (Supplementary Table S3). The rhizosphere fungal network consisted of 122 nodes and 626 edges, and the network diameter and clustering coefficient were 8 and 0.637, respectively (Supplementary Table S3). Both networks suggested that fungal community coexistence was greater than they would be separately in the phyllosphere and the rhizosphere. Some fungal families of Ascomycota had the highest overall betweenness centrality within phyllosphere and rhizosphere fungal networks (Figure 7 and Supplementary Table S4). Davidiellaceae and norank_o_Trichosphaeriales were the dominant fungal families in the phyllosphere, while Herpotrichiellaceae and Dothideomycetes played critical roles in the rhizosphere (Figure 7 and Supplementary Table S4).

### Functional Features of Phyllosphere and Rhizosphere Fungal Communities

FunGuild, a classification tool for fungi used to accurately define and reference assignments of trophic groups (Nguyen et al., 2016), was employed in this study to infer compositional variation in the functional groups of the phyllosphere and rhizosphere. Guilds identified during the three plant development stages are shown in Supplementary Figure S4. Animal pathogen/endophyte/plant pathogen/wood saprotrophs accounted for approximately 30.3–85.0% of all phyllosphere fungal OTUs detected. For the phyllosphere, dung saprotroph/plant saprotrophs were significantly higher during the seedling stage compared to the other stages, and the endophyte/plant pathogen yielded the highest relative abundance during the tiller stage (Supplementary Figure S4). Leaf saprotrophs were ubiquitous throughout the rhizosphere during plant growth processes (Supplementary Figure S4). For the rhizosphere, both endomycorrhizal/plant pathogen/undefined saprotrophs and undefined saprotrophs primarily subsisted during the seedling stage, while plant pathogens were generally higher during the mature stage (Supplementary Figure S4).

## Discussion

In copper tailings dams, the fungal community plays an important role, but it can potentially be affected by soil and plants traits (Jia et al., 2018c; Kong et al., 2019; Yang et al., 2020). However, most relevant studies only investigated a particular aspect related to differences in rhizosphere and/or non-rhizosphere communities (Jia et al., 2018a). Our study systematically investigated dynamic changes in rhizosphere and phyllosphere fungal communities during different plant development stages. We found distinct structural differences between phyllosphere and rhizosphere fungal communities among the different plant growth stages. The potential mechanism for this is the widely accepted assumption that plant leaves phenotypically differ among their different developmental stages, for which a subset of plant leaf migrants or colonizers are favored, consequently contributing to variation in fungal community composition (Leveau, 2019). In this study, phyllosphere and rhizosphere fungal community richness was both significantly higher during the tiller stage (Figure 1). The Shannon indexes of phyllosphere and rhizosphere fungal community were significantly higher during the tiller and seedling stage, respectively (Figure 1). This is in agreement with a previous eucalyptus study which showed that fungal distribution was distinct during different development stages, which could have been associated with the age of the host plant as well as surrounding environmental conditions (Jia et al., 2016). Chaparro et al. (2014) found that the rhizosphere microbial community of *Arabidopsis* differed significantly during the seedling stage compared to the other two stages. Fungi are capable of modifying the nutrient absorption kinetics of colonized plants during the tiller stage (Rho and Kim, 2017). Moreover, many studies have reported on the stronger fungal response to salt stress, invasive plant species, soil moisture, heavy metal pollution as well as other detrimental factors (Xiao et al., 2014; Kaisermann et al., 2015; Jia et al., 2019; Shen et al., 2019).

In this study, Ascomycota and Basidiomycota were the dominant fungal phyla groups within phyllosphere and rhizosphere samples, and this finding is in agreement with previous studies on other plant species (Ma et al., 2013). It has been reported that Ascomycota is the most abundant phylum found in rhizosphere communities (Hao et al., 2016) as well as being the dominant taxa in tropical grasslands in the country of Laos (Lienhard et al., 2014). We also found that Phaeosphaeriaceae and Mycosphaerellaceae, two fungal groups, were enriched during the seedling stage, and Ustilaginales were significantly enriched during the tiller stage in phyllosphere (Supplementary Figure S3). Phaeosphaeriaceae were commonly associated with plants as pathogens, though some are also saprotrophs and parasites on powdery mildews (Zhang et al., 2012), and Mycosphaerellaceae were usually assumed to be host-specific (Cortinas et al., 2006). Moreover, Ustilaginales included the plant pathogenic smuts that cause significant losses to crops worldwide (Martinezespinoza et al., 2002). This suggested that *B. ischaemum* plants could be resistant to pathogens in the primary stage of plant development. This could be that Ascomycota produces secondary metabolites that protect their hosts from pathogens (Blackwood et al., 2007; Rodriguez et al., 2009). Members of Basidiomycota, which is a phylum known to produce high amounts of lignin-modifying enzymes (LMEs), are believe to be the main decomposers in natural environments (Blackwood et al., 2007). Therefore, these two dominant fungal phyla could both play critical ecological adaptability in copper tailings dams, which will require further investigation in future studies.

Previous studies found that plant species, spatial locality, plant growth development, leaf structure, chemical composition, and secretion all affect phyllosphere microbial structure (Redford and Fierer, 2009; Hunter et al., 2010; Kim et al., 2012). Therefore, it stands to reason that plant traits are key factors that affect phyllosphere microbial community structure (Whipps et al., 2008). Our findings showed similar results, namely, that the phyllosphere fungal community structure was significantly affected by plant carbon. Moreover, Hypocreales, Xylariales, Polyporales, Corticiales, Russulales, Agaricostilbales, norank_c_Dothideomycetes, Cantharellales, Trichosphaeriales, Agaricales, and Sordariales all exhibited significant positive correlations to phyllosphere plant nitrogen. A possible explanation for this is that the plant nutrient content is determined by numerous factors, such as plant nutrient requirements and availability, nutrient absorption, and utilization efficiency and nutrient mobility (Marschener, 1998), and subsequently, may influence fungal community composition during this stage of development. Agaricales can degrade lignin and decompose litter (Morgenstern et al., 2008). Additionally, fungal community diversity and species richness were both higher in the rhizosphere compared to the phyllosphere, which is in agreement with an earlier study (Park et al., 2017). One study reported that tree traits can affect the patterns of fungal richness in leaves (Andrew et al., 2019). In our study, root properties were the dominate factor that influenced phyllosphere fungal diversity, while soil properties indirectly affected phyllosphere fungal diversity (Figure 6). This may result from the various effects that functional microorganisms have on plant growth during plant development, which will affect plant phenotypes and fungal community diversity (Spiering et al., 2006; Fitzpatrick et al., 2019; Russo et al., 2019).

Soil’s role in microbial rhizosphere structure is regulatory. In particular, soil pH, soil nutrients, and soil fertility are all considered important factors that affect rhizosphere microbial structure (Philippot et al., 2013). Our study showed that soil TS significantly affected the fungal community structure of the rhizosphere. He et al. observed that phosphorus (P) application treatments significantly changed the structure of the soil fungal community and resulted in a significant decrease in the community richness of fungi (He et al., 2008, 2016). It has also been reported that both fungal populations and fungal diversity in soil will be affected by soil pH, while high P application treatments can significantly change the structure of fungal communities (Živanov et al., 2017). Our study found that Agaricales, Sordariales, and Tremellales abundance was highly positively correlated to the pH level of the rhizosphere. Similarly, a previous study also found that there is a significant correlation between soil pH and the resident rhizosphere microbial community (Essel et al., 2019). Members of the class Agaricomycetes act as important decomposers, producing both hydrogen peroxide (H_2_O_2_) and enzymes, resulting in the degradation of complex plant compounds, such as cellulose and lignin (Kameshwar and Qin, 2016).

The role of fungal co-occurrence networks is important in revealing the interactions that exist among different species, such as through parasitism, competition, and mutualism (Zhou et al., 2011; Deng et al., 2012). In this study, the co-occurrence network identified certain keystone families, which demonstrate dynamical relationships between phyllosphere and rhizosphere fungal communities (Figure 7). Some fungal families of Ascomycota yielded the highest betweenness centrality values within the phyllosphere and rhizosphere fungal network (Supplementary Table S4). Davidiellaceae, norank_o_Trichosphaeriales, and norank_o__Pleosporales were the key fungal families found in the phyllosphere, while Herpotrichiellaceae and some unclassified families of Sordariales and Dothideomycetes all played critical roles within the rhizosphere (Figure 7 and Supplementary Table S4). It had previously been confirmed that certain low abundance taxa paradoxically play disproportionate roles in regulating ecological functions within various habitats (Zhang et al., 2018), which reveals the key roles that certain rare species play in ecosystems (Deng et al., 2016; Feng et al., 2017). The key to understanding fungal community development in environments under stress is to clarify the symbiotic relationships that exist among microbes that have successfully adapted to their host plants, which is also the case for plant health development (Andrews, 1992). In these key species, Davidiellaceae and Pleosporaceae fungal groups on leaves surface were driven by environmental climatic conditions (Gomes et al., 2018). Dothideomycetes were tolerant of harsh conditions, including nutrient limitation, high solar irradiation, fluctuating water availability, and osmotic stress (Egidi et al., 2014), and conditions that may exist in the phyllosphere microenvironment (Vorholt, 2012). This indicated that the key species of *B. ischaemum* fungal community had the ecological suitability in copper tailings dam. Moreover, Dothideomycetes were crucial to ecosystem functioning and global carbon cycling, as saprobes, they decompose cellulose and other complex carbohydrates in plant matter (Schoch et al., 2006; Ohm et al., 2012). Sordariomycetes and Dothideomycetes were the major classes involved in litter decomposition progresses, and these fungal were positively correlated with the contents of lignocellulose components during litter decomposition (Zhang et al., 2019). Hence, it is likely that these particular fungal played vital roles in decomposer community after leaf senescence in the damaged ecosystem. Furthermore, the positive correlations found between phyllosphere and rhizosphere fungal communities were relatively high, which indicated that microorganisms that inhabit this niche are mutualistic and not competitive in nature (Deng et al., 2016; Feng et al., 2017).

In the phyllosphere, the relative abundance of endophytes was highest during the tiller stage (Supplementary Figure S4); in the rhizosphere, the relative abundance of endomycorrhizae was highest during the seedling stage (Supplementary Figure S4). Plant properties and soil microbes can separately affect endophyte infection rates and endomycorrhizae by releasing exudates of low molecular weight (e.g., glomalin, amino acids, and organic acids) through external mycelia (Marschner and Timonen, 2005; Jia et al., 2017). Also, endophyte infection rates were typically higher during the tiller stage (Jia et al., 2017). Moreover, certain sugar exudates are considered critical to fungal community growth in the rhizosphere (Hooker et al., 2007). This could help explain the increased abundance of certain fungal groups in our study. Leaf saprotrophs were ubiquitous in the rhizosphere during plant growth processes (Supplementary Figure S4). A potential explanation for this is that saprotrophic fungi can inhabit fungal niches (Veresoglou et al., 2011) or compete with other fungi for carbohydrate resources (Baggi, 2000), which could cause an increase in abundance during the mature stage. We found that phyllosphere and rhizosphere fungal communities possessed distinct functional features. This indicated the ability of phyllosphere and rhizosphere microbial communities to be able to select specific functions throughout plant development (Chaparro et al., 2014).

Results from this study offer new insight into clarifying the dynamical relationships between phyllosphere and rhizosphere fungal communities during *B. ischaemum* development within a polluted copper tailings environment. However, there are still some limitations in our research. The plant heavy metal distributions and relationships between the heavy metals and fungal communities were also important in copper tailings dam. These experiments would be further studied in our future work. In brief, our study found that phyllosphere and rhizosphere microbial communities are favorable to the development and utilization of beneficial microbial communities at different stages of development, which could more effectively aid in the remediation and stabilization of land contaminated with copper tailings.

## Data Availability Statement

The datasets presented in this study can be found in online repositories. The names of the repository/repositories and accession number(s) can be found below: https://www.ncbi.nlm.nih.gov/, PRJNA605500.

## Author Contributions

TJ conceived and designed the experiments. YY analyzed the data. RW and TG performed the experiments. BC contributed new reagents. TJ wrote the manuscript. All authors contributed to the article and approved the submitted version.

### Conflict of Interest

The authors declare that the research was conducted in the absence of any commercial or financial relationships that could be construed as a potential conflict of interest.

The reviewer JL declared a shared affiliation with the authors to the handling editor at time of review.

## Footnotes

^1^http://rdp.cme.msu.edu/
