# Supplementary material for: Effects of Plant and Soil Characteristics on Phyllosphere and Rhizosphere Fungal Communities During Plant Development in a Copper Tailings Dam
Source: Front Microbiol. 2020 Sep 29;11:556002. doi: 10.3389/fmicb.2020.556002 (PMC7550642; doi:10.3389/fmicb.2020.556002)
Supplement: Supplementary file 1 [file Data_Sheet_1.pdf]

Effects of plant and soil characteristics on phyllosphere and rhizosphere fungal communities during plant development in a copper tailings dam

Tong Jia*, Yushan Yao, Tingyan Guo, Ruihong Wang, Baofeng Chai

Shanxi Key Laboratory of Ecological Restoration on Loess Plateau, Institute of Loess Plateau, Shanxi University, Taiyuan 030006, P.R. China;

*Corresponding author. Email: jiatong@sxu.edu.cn

Address: No. 92 Wucheng Road, Xiaodian district, Taiyuan, Shanxi 030006, P.R. China. Tel.: +86-155-1369-4458

Supplemental Material

Table S1

Mean values (± SE) of *Bothriochloa ischaemum* properties in copper tailings dam. Significant differences between sites (Duncan test, $P < 0.05$) are denoted with letters (a > b > c).

	Seedling	Tiller	Mature
TN_Leaf	3.213±0.758b	93.748±4.955a	5.199±1.844b
TC_Leaf	47.158±2.832b	45.319±0.313b	78.431±1.821a
C/N_Leaf	15.819±2.462a	0.486±0.028b	18.478±4.912a
TS_Leaf	1.009±0.702	0.157±0.009	0.519±0.184
TN_Sheath	0.817±0.031b	41.381±7.046a	1.329±0.085b
TC_Sheath	42.720±0.462a	33.346±0.656b	6.631±0.260c
C/N_Sheath	52.458±2.341a	0.854±0.148c	5.056±0.537b
TS_Sheath	0.180±0.022b	0.130±0.008b	0.802±0.093a
TN_Root	0.985±0.016b	9.392±3.120a	0.851±0.035b
TC_Root	44.337±0.032b	44.832±0.096a	15.330±0.277c
C/N_Root	45.038±0.693a	6.949±3.319c	18.089±0.871b
TS_Root	0.627±0.154a	0.244±0.009b	0.032±0.005c

Abbreviations mean total nitrogen (TN), total carbon (TC), total sulfur (TS), and the ratio of carbon and nitrogen (C/N); the unit of TN, TC and TS, $g \cdot kg^{-1}$.

Table S2

Soil chemical properties of copper tailings dam. Values represent mean with standard error in parenthesis. Significant differences between sites (Duncan test, $P < 0.05$) are denoted with letters (a > b > c).

	Seedling	Tiller	Mature
SWC (%)	0.841±0.036c	15.499±1.260b	86.162±0.213a
pH	8.203±0.020b	8.913±0.019a	8.113±0.029c
NH_4^+-N ($mg \cdot kg^{-1}$)	0.156±0.029bc	0.273±0.116ab	0.427±0.075a
NO_3^--N ($mg \cdot kg^{-1}$)	0.064±0.006c	0.220±0.019a	0.134±0.007b
NO_2^--N ($mg \cdot kg^{-1}$)	0.006±0.001b	0.008±0.001b	0.011±0.002a
TN ($g \cdot kg^{-1}$)	0.082±0.001a	0.029±0.003c	0.047±0.005b
TC ($g \cdot kg^{-1}$)	6.017±0.919a	0.627±0.018b	1.033±0.041b
C/N	73.516±11.817a	21.681±1.164b	22.058±1.474b
TS ($g \cdot kg^{-1}$)	0.459±0.294a	0.065±0.002b	0.144±0.017ab
ST (℃)	23.900±0.473c	28.833±0.698a	26.867±0.484b
Salinity ($mg \cdot L^{-1}$)	0.000±0.000c	14.333±3.844b	31.667±1.202a
EC ($\mu s \cdot cm^{-1}$)	0.000±0.000c	26.667±7.219b	57.667±1.667a

Abbreviations mean soil water content (SWC), ammonium nitrogen (NH_4^+-N), nitrate nitrogen (NO_3^--N), nitrite nitrogen (NO_2^--N), total nitrogen (TN), total carbon (TC), total sulfur (TS), the ratio of carbon and nitrogen (C/N), soil temperature (ST), and electrical conductivity (EC).

Table S3

Network properties obtained through network analysis from phyllosphere and rhizosphere fungal communities.

	Nodes	Edges	Average Degree	Network Diameter	Clustering Coefficient	Average Path Length
Phyllosphere	82	416	10.146	9	0.685	3.218
Rhizosphere	122	626	10.262	8	0.637	3.574

Table S4

Dominant keystone species from phyllosphere and rhizosphere fungal community structures.

	Phylum	Family	Betweenness centrality
Phyllosphere	Ascomycota	Davidiellaceae	529.80
	Ascomycota	norank_o_Trichosphaeriales	404.33
	Ascomycota	norank_o_Pleosporales	388.47
	Ascomycota	Trichocomaceae	325.55
	Ascomycota	Montagnulaceae	280.90
	Ascomycota	Myxotrichaceae	268.00
	Ascomycota	Stictidaceae	263.18
	Ascomycota	Dothioraceae	262.64
	Ascomycota	Mycosphaerellaceae	216.06
	Basidiomycota	Marasmiaceae	196.84
Rhizosphere	Ascomycota	unclassified_o_Sordariales	775.03
	Ascomycota	unclassified_c_Dothideomycetes	749.90
	Ascomycota	Herpotrichiellaceae	622.33
	Ascomycota	unclassified_p_Ascomycota	566.71
	Ascomycota	Lasiosphaeriaceae	552.13
	Ascomycota	norank_p_Ascomycota	519.77
	Basidiomycota	unclassified_c_Agaricostilbomycetes	532.27
	Basidiomycota	unclassified_o_Sebacinales	530.93
	Basidiomycota	Ustilaginaceae	502.57
	Basidiomycota	Typhulaceae	452.00

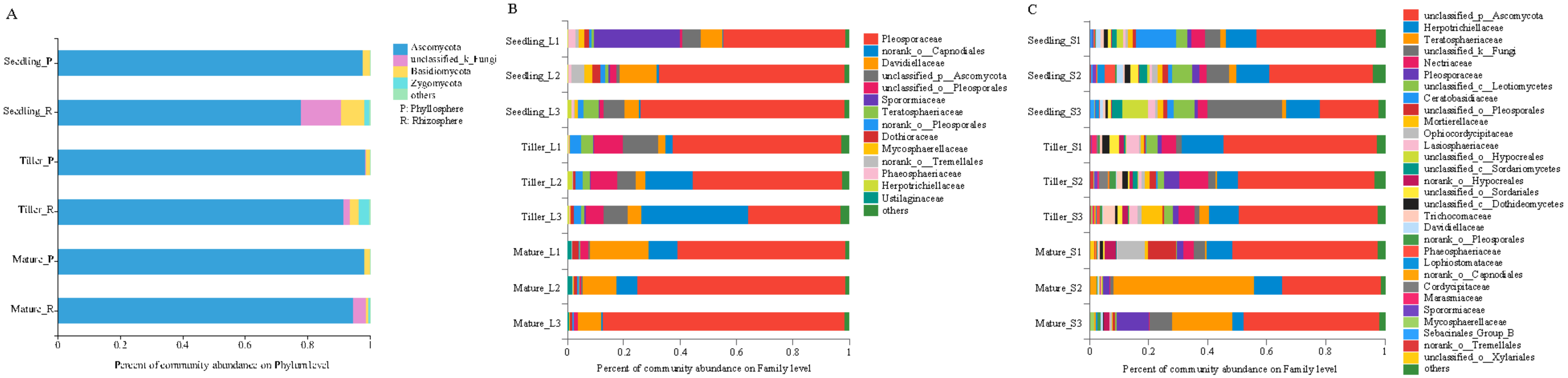

FIGURE S1 Relative abundance of fungal phyla (A), dominant phyllosphere (B) and rhizosphere (C) fungal community families (with average relative abundance > 1%) among different plant growth stages.

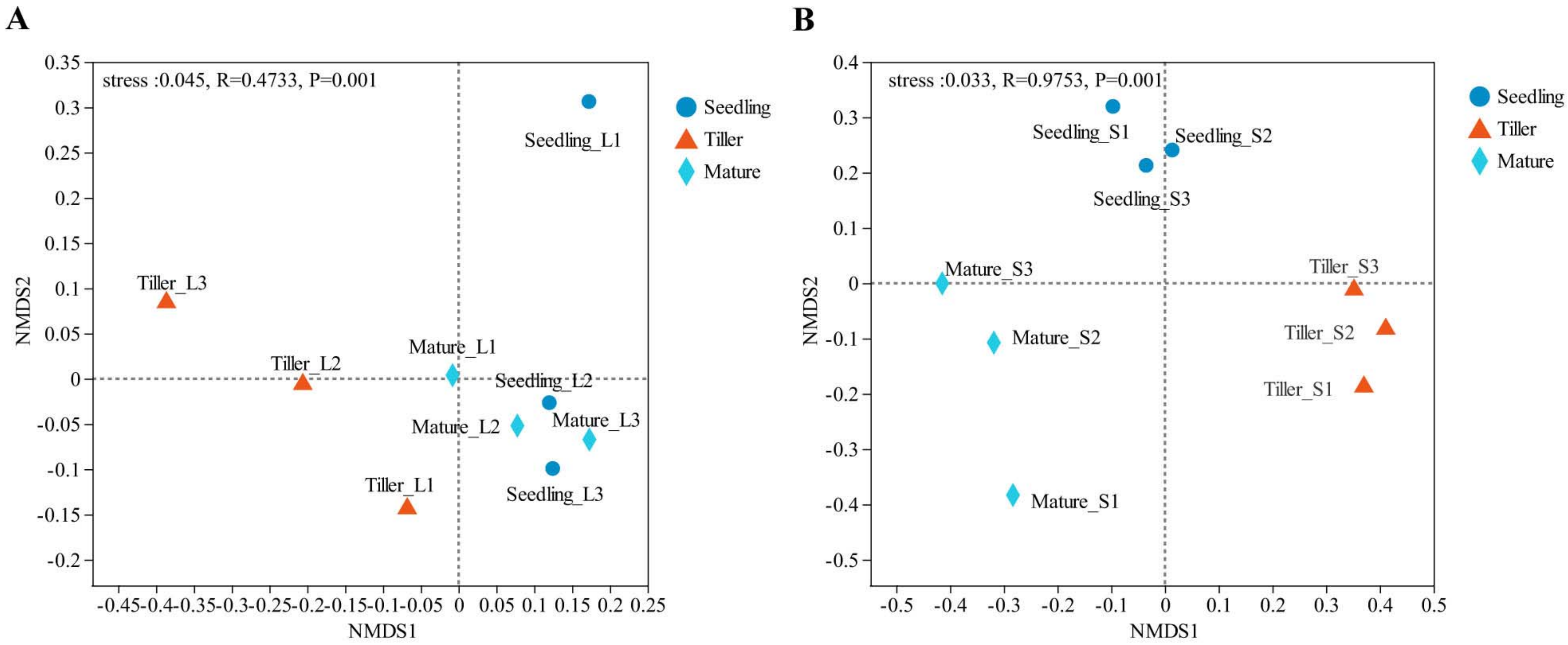

FIGURE S2 Non-metric multidimensional scaling (NMDS) of phyllosphere (A) and rhizosphere (B) samples among plant development processes based on the relative abundance of fungal OTUs.

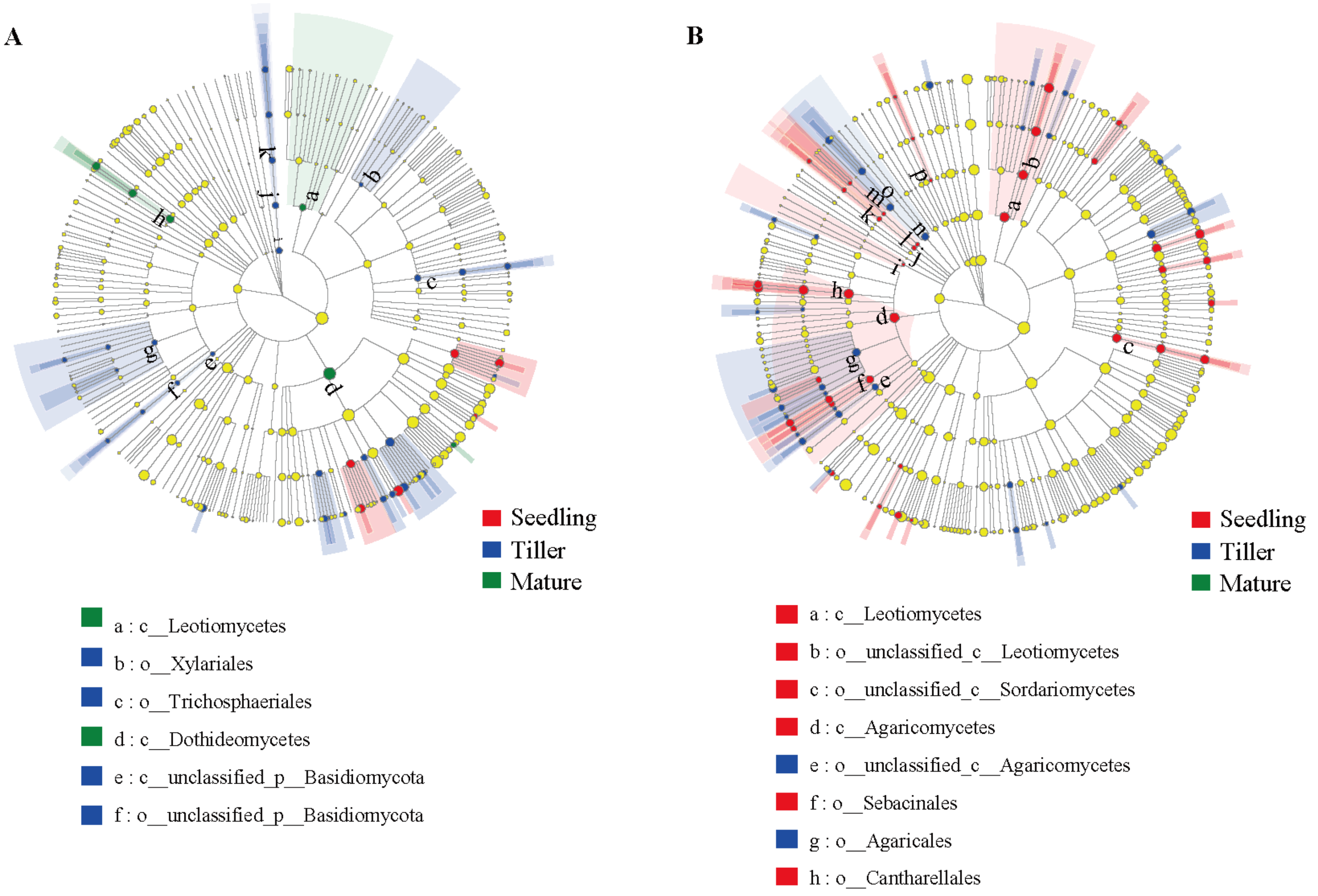

FIGURE S3 Cladogram showing the phylogenetic distribution of phyllosphere (A) and rhizosphere (B) fungal communities from different development stages. Different-colored regions represent different constituents (red, seedling; green, mature; blue, tiller. Circles indicate phylogenetic levels from phylum to genus. The diameter of each circle is proportional to the abundance of the group.

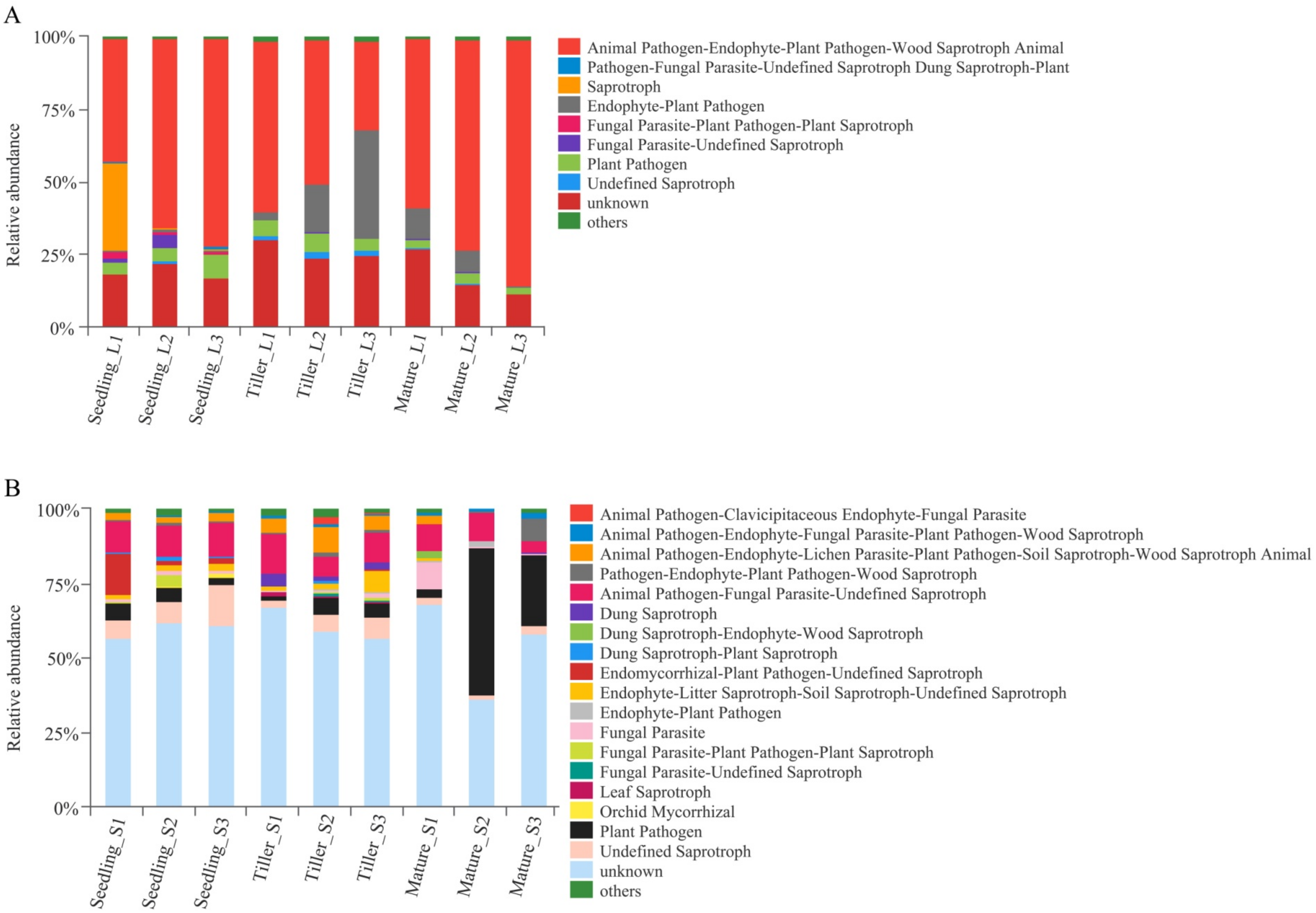

FIGURE S4 Variations in composition of phyllosphere (A) and rhizosphere (B) fungal functional groups inferred by FunGuild during plant growth processes.
